# Monitoring Immune Cell Function Through Optical Imaging: a Review Highlighting Transgenic Mouse Models

**DOI:** 10.1007/s11307-021-01662-5

**Published:** 2021-11-04

**Authors:** Chintan Chawda, Roisin McMorrow, Natasa Gaspar, Giorgia Zambito, Laura Mezzanotte

**Affiliations:** 1 Department of Radiology and Nuclear Medicine, Erasmus MC, Rotterdam, The Netherlands; 2 Department of Molecular Genetics, Erasmus MC, Rotterdam, The Netherlands; 3 Percuros B.V, Leiden, The Netherlands

**Keywords:** Transgenic mouse models, Immune cells, Fluorescence, Bioluminescence, Intravital imaging

## Abstract

Transgenic mouse models have facilitated research of human diseases and validation of therapeutic approaches. Inclusion of optical reporter genes (fluorescent or bioluminescent genes) in the targeting vectors used to develop such models makes *in vivo* imaging of cellular and molecular events possible, from the microscale to the macroscale. In particular, transgenic mouse models expressing optical reporter genes allowed accurately distinguishing immune cell types from trafficking *in vivo* using intravital microscopy or whole-body optical imaging. Besides lineage tracing and trafficking of different subsets of immune cells, the ability to monitor the function of immune cells is of pivotal importance for investigating the effects of immunotherapies against cancer. Here, we introduce the reader to state-of-the-art approaches to develop transgenics, optical imaging techniques, and several notable examples of transgenic mouse models developed for immunology research by critically highlighting the models that allow the following of immune cell function.

## Introduction

Immune cell function has always been found to be influenced by the tissue microenvironment. An *in vivo* immune response relies on the presence of a tissue environment that allows for pathogen entrapment, processing and presenting of antigens, [1–3, 13] immune cell priming [4–6], or modulation. [7–13] On the contrary, in an *in vitro* setting, immunogenic responses are proportional to the degree of the administered stimuli or environmental cues that they are exposed to. Interestingly, the expected outcomes noted in tumor immunology studies can show contrasting results when tested *in vivo*, [14–16] the primary reason being the absence of innumerable peripheral or tissue-associated immune-modulatory entities. Thus, the true nature of interactions and induced response within an immune-oncological setting can be reported precisely when studied *in vivo*.

Using transgenic (TG)^1^ mice as a model for biomedical research has its advantages. As highlighted in the 2002 Mouse Genome Sequencing Consortium, mice and humans share "78.5 %" of amino acid identity within their orthologous genes, supported by a corresponding "90 %" of genomic synteny between them. [17] Secondly, the possibility to selectively cross TG mice, which leads to conditional gene inactivation, allows the progeny to express the desired gene of interest. Genetic manipulations during the early developmental stage, may it be gene insertion, deletion, or modification, render the previously modulated gene resistant against immune recognition and activation in the later adult stage of TG mice. This ensures for stable foreign gene integration or expression in future generations.

Real-time *in vivo* imaging has facilitated the visualization of distinct pro-tumoral or anti-tumoral behavior of immune cells in mouse models of cancer. To state a few are tumor-associated macrophages (TAM) for silencing tumor-infiltrating CD8 + T cells, [18] natural killer (NK)^2^cells clearing pulmonary metastasis of anaplastic thyroid cancer cells, [19] the pro-metastatic role of neutrophils, [20] and immunosuppressive activity of tumor-derived dendritic cells, [21] revealing the pathway activating a humoral immune response in a melanoma model [22] and contribution of stromal cells in navigating metastasis. [23–25] Due to their small size, whole-body optical imaging of the TG mice is possible as well as visualization of a low number of cells. [26] Notably, bioluminescence and fluorescence *in vivo* imaging, along with optical tomography modalities, rendered *in vivo* imaging to be highly sensitive, [27–29] with an additional advantage of being able to employ safe non-ionizing radiation. Lastly, along with the abovementioned characteristics, optical imaging is considered a medium throughput and cost-effective modality compared to magnetic resonance imaging (MRI) or positron emission tomography (PET). In this review, we highlight transgenic mouse models that, by means of *in vivo* imaging, contribute to the understanding of immune cell function in health and disease.

## Reporter Gene Knock-in Transgenic Mouse Models

### Constitutive Knock-in

**Fig. 1.**
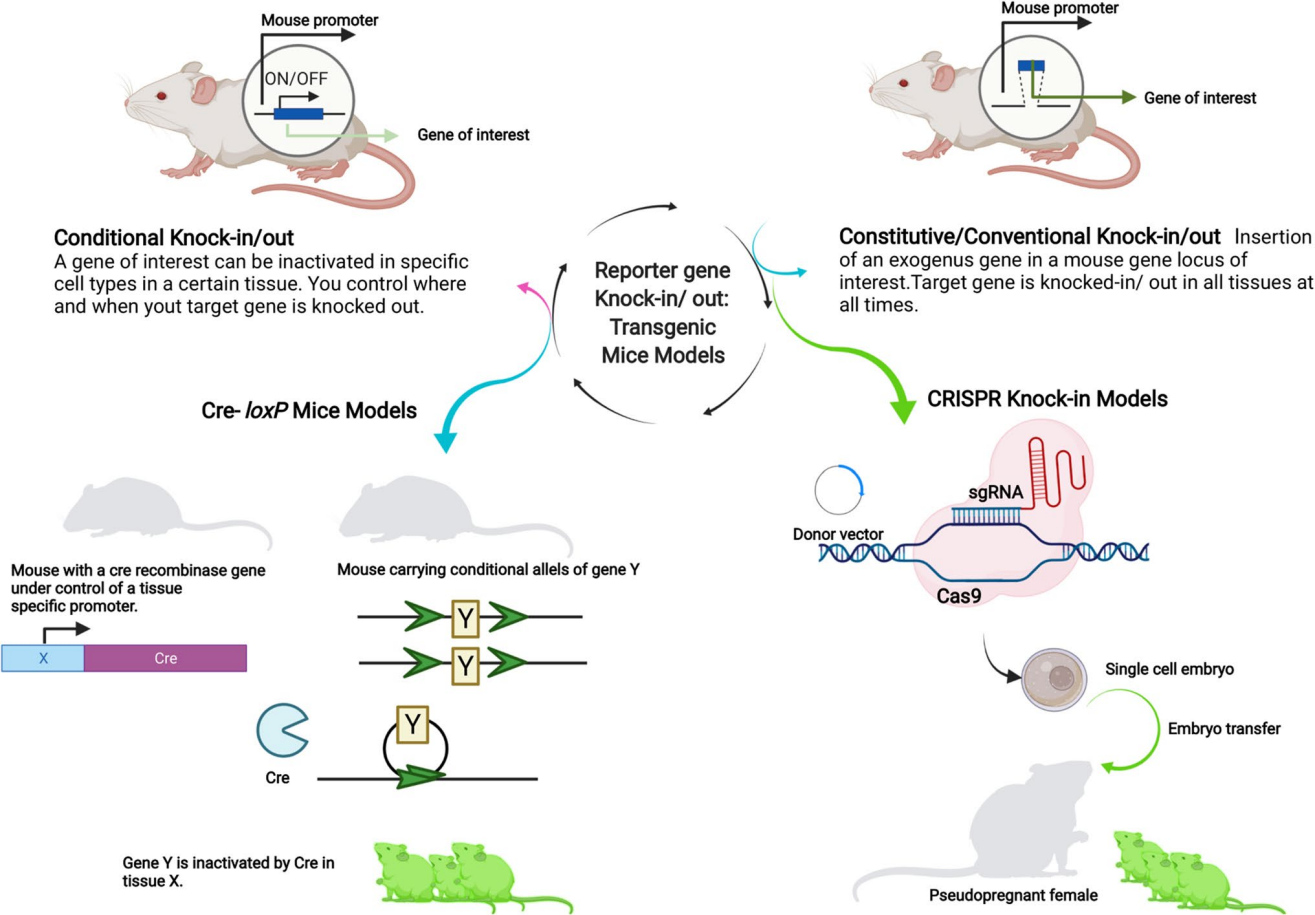
State-of-the-art technologies and approaches for development of transgenic mouse models. Constitutive knock-in mouse models are generated using a target vector carrying gene of interest into the early mouse embryonic cells. Within the mouse genome, the integration of the target vector might occur randomly or through induced homologous recombination. Conditional knock-in mouse models where expression of gene of interest occurs at a specific time or tissue are involved in the majority of cases using the Cre-*loxP* system. Cyclization recombinase (Cre) recognizes specific DNA recognition sites (LoxP sites) and catalyzes a site-specific recombination event between those two sites.

TG mouse models represent ideal models to study basic cancer research and translational oncology, which has been reviewed meticulously in previous studies. [30–32] Such targeted mouse transgenesis mirrored features involved in immune responses, [33–35] cancer progression, [36–38] stroma, [39, 40] and pharmacokinetics [41–45] as observed in humans. A comprehensive overview of different techniques involving TG mouse generation is discussed in the work of Tratar et al. [46] Out of multiple techniques for generating TG mouse models, here we discuss transgenesis techniques involved in generating gain of function mutation. Briefly, constitutive knock-in mouse models are generated by homologous recombination of a targeted vector or due to random integration of a target cassette within the mouse genome upon transfection (Fig. 1). This modified vector is then introduced into the early embryonic cells via microinjection or viral transduction. [47–50] Random integration might elevate the chances of unwanted gene silencing or unregulated gene expression, reducing the desirability of this model. On the other hand, it may result in multiple integration sites and higher expression of the reporter. The vectors usually contain a strong constitutive promoter or a tissue-specific one to restrict expression in certain organs. More recently, there has been an increased interest in a gene editing-based knock-in approach that utilizes the CRISPR-Cas^3^ (clustered regularly interspaced short palindromic repeats-cellular apoptosis susceptibility) system. The CRISPR-Cas system involves a nucleoprotein complex which consists of a single-guide RNA (sgRNA) sandwiched within a CAS nuclease protein (most commonly Cas9) or co-transfecting multiple sgRNA along with plasmids expressing CAS [51] or Cas9 mRNA [52] into early embryonic cells. This sgRNA is specific and complementary to the gene of interest. Within the host nuclei, this system scans the genome locus in search of the complementary sequence, which, upon recognition by Cas9 nuclease, makes a double-strand break in a specific protospacer adjacent motif (PAM) site near the complementary sequence, allowing gene editing. [53, 54] These DNA double-strand breaks are further repaired by non-homologous end joining (NHEJ) or homology-directed repair (HDR) pathway. The latter is necessary for gene addition through homologous recombination and the generation of knock-in models (Fig. 1). The reporter genes are then expressed at the endogenous level if the sequence is inserted in tandem with another endogenous gene. On the other end, a cassette can be knocked-in to a specific locus, like the Rosa26 (reverse orientation splice acceptor) locus. [55] Kersten et al. [30] underlined different subtypes of CRISPR-Cas9 systems developed by multiple groups, discussing the different CRISPR-Cas9 subtypes for inducing gene-specific point mutations, translocations, and insertions. Due to its high efficiency, target specificity, versatility, and cost-effectiveness, CRISPR-Cas9 has rapidly become the most favored approach for generating TG mice. [56]

### Conditional Knock-in

**Fig. 2.**
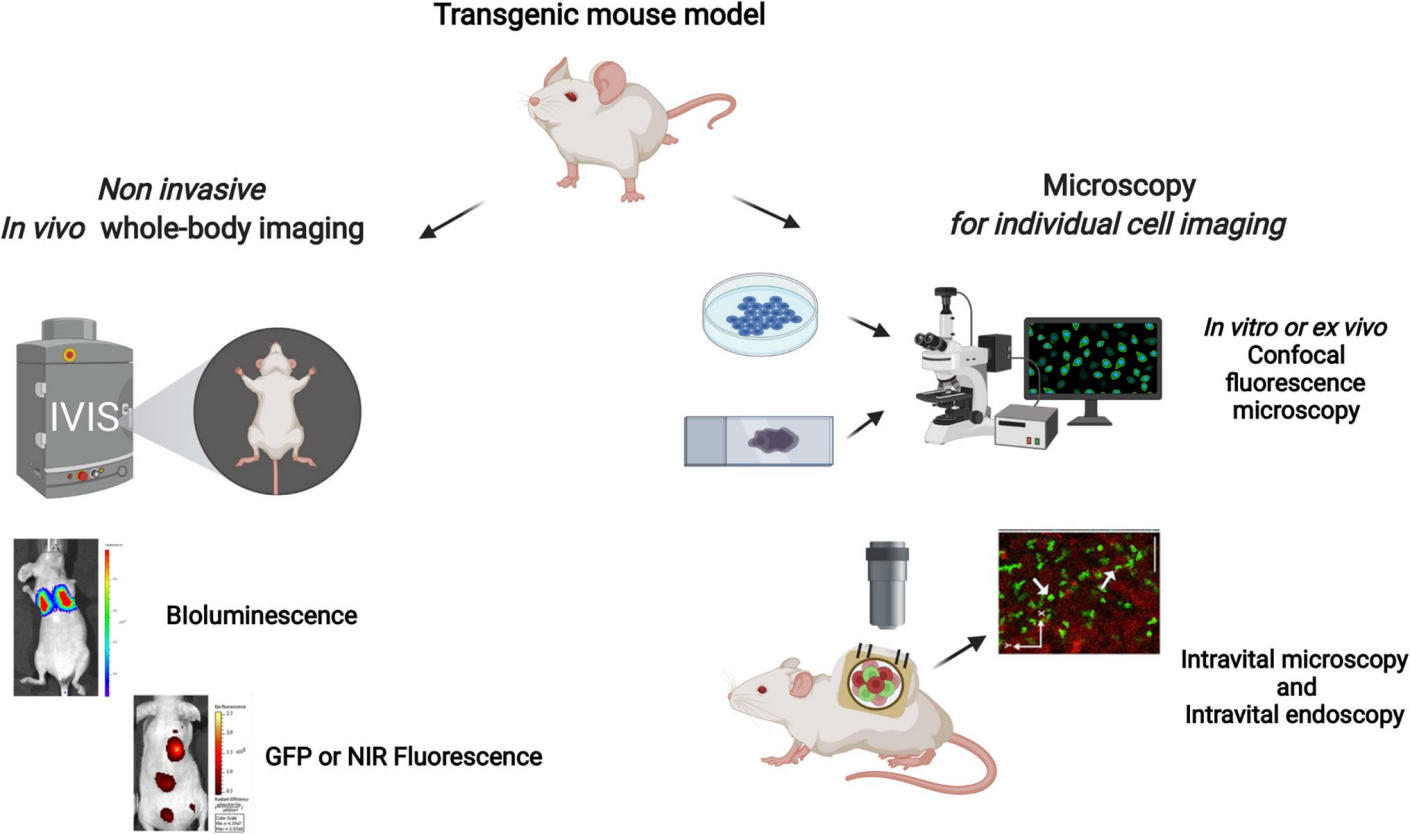
Different optical imaging techniques for *in vivo* imaging.

In order for a more controlled, site-directed manipulation, this requires a conditional knock-in approach. Conditional manipulation involves the Cre-*loxP* system. Cyclization recombinase (Cre)^4^ recognizes specific DNA recognition sites (LoxP sites) and catalyzes a site-specific recombination event between those two sites. Other specific recombinases are flipase (Flp) and D6-specific recombinase (Dre), which demonstrate lower recombination efficiency compared to Cre. In a common application, the gene of interest to be silenced is floxed by *loxP* gene segments present in the first parent mice. At the same time, the Cre recombinase gene is inserted downstream of a tissue-specific promoter in the second parent mice (Fig. 2). Pups generated from crossing parent mice acquire a tissue-specific promoter-driven Cre recombinase. Expressed Cre then tracks down *loxP* (locus of X-over P1) sites. Identification of the *lo*x*P* sequence triggers recombinase activity and so cleaving the entire *lo*x*P-*floxed gene segment. This gene removal referred as conditional silencing makes the progeny TG. [57] As an alternative, a *loxP*-STOP-codon-*loxP* can be inserted upstream of the gene of interest. That tissue-specific, promoter-driven expression can remove the floxed STOP codon. This in turn would allow for the gene of interest to be expressed, which was earlier unexpressed in the parental generation. [57] To highlight, an improved Cre recombinase mutant (iCre) being a codon-optimized version displays improved expression and accuracy. [58] A more regulated version of Cre-*loxP* system, namely Cre-ERT, was developed by Feil et al. [59] It consists of a Cre recombinase integrated with a mutated ligand-binding domain of the estrogen receptor. Only after administration of tamoxifen (estrogen analog) and its binding to the altered Cre-recombinase can it pass through the nuclear envelope and spatiotemporally perform targeted restriction. [59, 60]

## *In Vivo* Optical Imaging

*In vivo* optical imaging relies on the detection of luminescent signals reaching the surface of the imaging object using sensitive light detectors as charge-coupled device (CCD) cameras or photomultiplier tubes. [61, 62] In most of the applications, photons are generated by a chemiluminescent reaction, as in the case of luciferase enzymes, or by a fluorescent protein. In fact, both the luciferases and fluorescent proteins can be utilized as reporter genes for developing transgenic mice making them available for imaging purpose. Note that reporter genes for other imaging modalities exist as for examples for radionuclide imaging (from which Cerenkov luminescence can be generated depending on the radiometal used), optoacoustic imaging, and magnetic resonance imaging but their use for the development of transgenic mice is rather limited. The main difference between fluorescent proteins and luciferases is that fluorescent proteins require an excitation light source while luciferases catalyze the reaction of a substrate and cofactors emitting photons as a reaction product. [61, 62] Since the light emission differs in intensity between fluorescence and luminescence, the general setup for fluorescent and bioluminescent signal acquisition might differ too, in order to optimize the resolution or detection sensitivity. [63] In addition, multiple lasers assisted excitation and light collection using emission filters paves the way to achieving multicolor imaging. [63, 64] *In vivo* imaging can be performed as a whole-body optical imaging or targeting a specific location through intravital imaging.

### Whole-Body Imaging

Whole-body optical imaging is non-invasive and employs sensitive cameras for light detection. They are mounted on the top of a black box which contains a heat-regulated platform for animals to be placed. In order to perform fluorescence, the instruments are equipped with lasers or with multiple optical filters that are placed in front of a lamp and are used as a light excitation source. [63] In addition to that, a filter wheel in front of the detector is inserted for selecting optimal emission wavelengths. Multiple whole-body optical imaging instruments allow detection of fluorescence or chemi-/bioluminescence. Whole-body imaging can reach 20-µm resolution and a degree of sensitivity that allows single cells to be imaged. However, it is mainly employed to visualize multiple cells expressing fluorescent or bioluminescent reporter proteins. [62, 63] Cells localized in both superficial and deep tissue can be imaged with enough resolution depending on the brightness of emitted signals, on the ability to excite the fluorescent proteins at a depth of several millimeters, and lastly on the background light generated. In general, due to high absorption and scattering of UV/visible light by mammalian tissue, green and red photons can be detected only superficially acting as a drawback for its use in deep tissue imaging. In contrast, near-infrared-emitting proteins can be visualized in deeper organs with a compromise of image resolution. [61, 62]

### Intravital Imaging

Intravital imaging is an invasive optical imaging technique which employs mountable transparent imaging window chambers that can be fixed in different parts of the animal. [65–67] The imaging window allows optical fibers to excite and convey light to the detectors. [68] With these settings, intravital imaging achieves cellular or even sub/cellular resolution *in vivo*. [65, 67] The main advantage of intravital imaging is that intercellular interactions can be visualized and studied in the original microenvironment. Intravital microscopy (IVM)^5^ is performed using several light microscopy techniques like wide-field fluorescence, multiphoton, or confocal. Furthermore, operative robustness of window chamber allows imaging to be performed in the same animal repeatedly. On the other hand, intravital endoscopy, as confocal laser scanning microscopy which employs an optical fiber, is a minimally invasive technique that has enabled *in vivo*, real-time microscopic tissue visualization. [68, 69] The flexibility of the optical fiber makes the confocal microscopy accessible to the region to image *in vivo* without losing its optical sectioning and high resolution. Intravital imaging has been extensively adopted for imaging fluorescent cells in transgenic mice and recently has also been adapted to image low light generated by chemiluminescence. [70] On the other side, respiration or heartbeat-induced periodic tissue moment might compromise the resolution and cause image shift of the examined region. [71]

### Cryo-fluorescence Imaging and Tomography

As most of the TG mouse models possess fluorescent reporter genes, performing ex vivo imaging can illuminate the morphofunctional features of the imaged tissue. One such example of ex vivo imaging is cryo-fluorescence imaging. In this modality, vitrified (ultra-rapidly cryopreserved) and sectioned tissues are imaged using fluorescent microscopy. To add, 2D (dimensional) ex vivo data can be visualized in 3D using cryo-fluorescence tomography. This can be achieved by sectioning an entirely vitrified mouse in a frontal, transverse, or sagittal plane followed by fluorescent imaging of each tissue section. Imaged tissue sections are then stacked and aligned using software to develop a whole-body 3D image of the body. The tissue specimen being cryo-immobilized is advantageous as, first, it exhibits a near-native tissue state, rendering ultrastructural resolution at a cellular level when tagged by fluorescent molecules. Second, the fluorophores being frozen have a reduced tendency towards photobleaching, thus increasing the half-life of the fluorophore and emitted photons. [72] An overview of prospects and limitations surrounding cryo-fluorescence imaging can be found in the review of Kaufmann et al. [73]

## Transgenic Mouse Models for Imaging Immune Cell Function

The last two decades have provided a significant research output in the field of non-invasive, real-time optical imaging of model organisms. While the equipment, techniques, and probes were upgraded for *in vivo* imaging, so were the gene manipulation techniques involved in the generation of TG reporter mice. There is an abundance of available TG reporter mouse models that are used for imaging different immune cell subsets. To highlight, some examples are the developments of transgenic mice that are useful for cell lineage tracing or cell trafficking related to monocytes, [74–77] regulatory T cells, [78–80] macrophages, [81] microglia, [82] and interestingly megakaryocytes and platelets. [83] Although those TG reporter models are valuable for *in vivo* imaging, in this review, we will focus on examples of TG mouse models that report specific immune cell function or report the function of an immunogenic modulator by means of *in vivo* imaging (compiled in Table 1).

**Table 1.** Brief overview of transgenic mouse models represented in this review

Designated transgenic reporter mouse model	Function of the reporter transgen in the respective mouse model	Reference
B6.Cg-Tg (Itgax- EYFP1) Mnz/J mice	Spatiotemporal visualization of CD11c + dermal dendritic cell (D.C) during transcutaneous immunization	[117]
B6.CXCR6^GFP+^ mice	Visualize temporal and migrational characteristics of immunostimulant-induced natural killer T cells (iNKT)	[88]
BLITC mice	Visualizing T cell localization and activation status	[89]
*Ccl20-Luc* reporter mice	Visualize tissue-specific expression profile of *Ccl20* and monitor immune activation when challenged with stimulant, flagellin	[95]
CD11c-EYFP reporter mice	Visualizing temporal kinetics of mature and immature dendritic cells in lymph node compartment	[107]
CXCR4^fl/fl^-ZBTB16^cre/+^ reporter mice	Studying importance of CXCR4 receptor interaction with cytokine CXCL12 on skin-resident natural killer T cells for immune activation and infiltration	[96]
*Cx3cr1*^*gfp/*+^*Flt3L*^*−/−*^ reporter mice	Visualization of bone marrow-derived monocyte migration from B.M to the site of infection	[109]
Foxp3-eGFP*DsRed reporter mice	Distinctive visualization and periodic monitoring of naïve and effector T cells infiltrating implanted allograft	[84]
Foxp3.LuciDTR reporter mice	Visualizing homeostatic regulating capability of regulatory T cells	[87]
Gammaglow reporter mice	Long-term visualization of IFN-γ expressed in response to infection, tumor growth, and autoimmune induction	[97]
*hIL6*-BAC-*Luc* (WIM-6) reporter mice	Visualization of *IL6* expression dynamics in induced inflammation and anti-inflammatory studies	[102]
Iba1-EGFP reporter mice	Spatiotemporal visualization of microglia during embryonic development	[113]
IL-1β IDOL reporter mice	Visualization of *IL-1β* expression dynamics in systemic and tissue-specific inflammation studies	[101]
*il12p40*-eYFP (yet40) reporter mice	Visualizing migration of *il12p40*-expressing bone marrow-derived dendritic cells into draining lymph nodes	[99]
*lys*-EGFP-*ki* reporter mice	Observing spatiotemporal dynamics of hematogenous macrophages and microglial macrophages within spinal cord lesion	[103]
LysM-LG reporter mice	Visualization of activated tumor-associated macrophages	[110]
TbiLuc transgenic reporter mice	Visualizing T cell localization and activation status	[91]
Thy1-CFP//LysM-GFP//CD11c-EYFP (triple transgenic) reporter mice	Visualize myelomonocytic cell subset migration and interaction within spinal cord lesion (SCL)	[105]

### Transgenic Mouse Models for Monitoring T Cell Function

Non-invasive *in vivo* imaging not only captures the uninfluenced intercellular interactions but also indicates the cellular kinetics and localization of the said cells. The latter might be of great value when studying immune cell infiltration in tissue grafts. To accomplish this, a transgenic color-coded T cell reporter mouse model was developed by Fan et al. [84]

The transgenics involved crossing of Foxp3-EGFP^6^ knock-in regulatory T cell mice [85] with DsRed^7^-TG mice. [86] The pups generated expressed Foxp3-EGFP*DsRed transgene. [84] Optically, the authors could distinguish the allograft infiltrated T cell subset using endoscopic confocal microscopy. Intravital examination was conducted in the recipient mouse post-transplantation of naïve regulatory T cells (nTreg)^8^ and Foxp3^−ve^ effector T cells (Teff)^9^ following islet engraftment. The former nTreg isolated from Foxp3-EGFP knock-in mice was seen (DsRed^−ve^ GFP^+ve^), while the other, Teff isolated from DsRed-TG mice, was observed (DsRed^+ve^ GFP^−ve^). Lastly, when the engrafted mice, transplanted with Teff (red) and nTreg (green), were assessed under a tolerance-inducing protocol (treated mice), antigen activated nTreg reprogrammed to Foxp3 and DsRed double-positive iTreg (Foxp3^+ve^ GFP^+ve^ DsRed^+ve^), which were identifiable as yellow cells. On the other hand, periodic examination of the tissue grafts with a minimally invasive endomicroscope revealed an exponential increase in Teff (red) cells with no significant nTreg (green) infiltrating engrafted islets.

In order to reveal the homeostatic regulating capabilities of Tregs, Suffner et al. developed bacterial artificial chromosome (BAC)^10^ TG mice, Foxp3.LuciDTR. [87] The transgenics involved the generation of a construct that incorporated EGFP, human DTR, and click beetle green luciferase (CBG99)^11^ cDNA. This series of genes was driven by an upstream Foxp3 promoter sequence. The cDNAs were separated by a self-cleaving 2A peptide sequence, for independent translation and protein folding. Foxp3 promoter drives the expression of EGFP fluorescence, CBG99 bioluminescence (in the presence of luciferin), and lastly DTR expression. The encoded DTR, when expressed, translates to a diphtheria toxin (DT)-specific receptor, which, in the presence of DT, can ablate regulatory T cells within those TG mice. This was evident by a significant reduction in emitted bioluminescent signal. [87]

Furthermore, to investigate the emigrational dynamics of induced natural killer T cells (iNKT) using real-time IVM, Thanabalasuriar and colleagues [88] developed the B6.CXCR6^GFP+^ TG mice. For IVM, TG mice were surgically implanted with a vacuum-stabilized lung optical window. While the lung vasculature was labeled red with tetramethylrhodamine isothiocyanate (TRITC)-dextran, iNKT cells were visible as green by marking them through PBS-57 ligand-conjugated CD1d tetramer. Using this model, the authors could not only distinguish temporal information but also note the CXCR6^GFP+^ cell (iNKT) emigrational characteristics, such as crawling, tethering, or being stationary in and around the lung vasculature. To recapitulate a more *in vivo* like iNKT cell activation and migrational dynamics, the authors challenged the mice with alpha-galactosylceramide (synthetic glucolipid immunostimulant). The iNKT activation gave an insight into how these cells crawled, extravasate, and changed their morphology with respect to time.

Szyska et al. applied a bioluminescence reporter system in order to generate dual-luciferase mice that reported T cell function and localization. They named it bioluminescence imaging of T cell (BLITC) TG mice. [89] These TG mice were a cross product between ChRLuc mice [90] and nuclear factor of activated T cell (NFAT)^12^-driven click beetle red luciferase (CBR)^13^ TG mice. The former ChRLuc mice possessed sustained expression of *Renilla luciferase* (RLuc). The latter NFAT-CBR mice comprised an artificial NFAT-responsive, promoter-driven, CBR construct which also possessed necessary viral elements allowing stable insertion into the C57BL/6NCrl mouse genome. Crossing the abovementioned TG mice generated RLuc^+/+^ NFAT-CBR^+/+^ pups. Sustained RLuc expression in the presence of coelenterazine substrate generated a bioluminescent signal exhibiting T cell migration. On the other hand, stimulant-driven T cell activation induced NFAT expression, giving T cells a CD8-positive status. Thus, NFAT induction in turn allows CBR expression, which, in the presence of a D-luciferin substrate, emits bioluminescence of a specific wavelength, allowing activated T cells to be recognized.

As a result, the optimal traceability of these dual-luciferase T cells was only observable when adoptively transferred in recipient mice, the reason being extensive RLuc expression from precursor cells with coelenterazine, [90] associated in a high background signal in BLITC mice.

In the following year, a TG reporter mouse, named TbiLuc, was generated by Klenovink et al. [91] Strategically, the TG modulation involved a bicistronic construct. The construct comprised two cassettes separated by an insulator. The first cassette contained a red-emitting firefly luciferase (PpyRE9) downstream of three repetitive NFAT response elements. The second cassette consisted of CBG99 luciferase mutant downstream of the human CD2 promoter. Microinjecting this construct into the pronuclei of a fertilized CBA^*^C57BL/6 mouse oocyte produced TbiLuc pups. Functionally, these dual luciferases were unlike those in BLITC mice. The human CD2 promoter drives CBG99 expression, which, in the presence of D-luciferin, allows for T cell localization, while the induced NFAT response element driving PpyRE9 luciferase expression, in the presence of luciferin substrate (CycLuc1), generates a distinct luminescence signal that distinguishes activated T cells and NK cells apart from their naïve T cell subtype.

### Transgenic Mouse Models to Monitor: Chemokines

Cytokines are a group of cell signaling molecules that play an important role, not only as key inflammatory modulators, but by also regulating metabolism, cell proliferation, angiogenesis, and wound healing. By binding to their specific receptors, they act as an agonist (relaying a stimulatory signal towards the nucleus) or as an antagonist (relaying an inhibitory signal) affecting cell metabolism and functionality. Furthermore, they can regulate cell activity in a paracrine (one cell communicating with another) or in an autocrine (self-stimulating cell) manner. Sharing the same origin, interleukins (meaning *inter*, within; and *leukin*, leukocytes) are a group of cytokines that focus mainly on immune and inflammatory stimuli while chemokines are cytokines that mainly trigger chemotaxis and attracting leukocytes to the cellular source of origin. Chemokine families are distinguished based on the number of conserved cysteine residues: one being the C-X-C family in which two cysteines are bridged by *n*-number of amino acids, while the other, the C–C subfamily, has both cysteines adjoining each other. [92] Screening the human genome revealed 44 chemokines and 23 chemokine receptors, as shown by Nomiyama et al. [93] Lastly, interferons (INF)^14^ are groups of cytokines that are released from a virally infected cell (INF-α and INF-β as type-1 INFs) or secreted by T cells, macrophages, or NK cells (IFN-γ) in response to inflammatory cues. [94]

In order to understand the expression profile of specific chemoattractant and how they modulate immune cell function, chemokine-specific TG mice [95, 96] were developed. To begin with, Crispo and colleagues generated *Ccl20* reporting TG mice, namely, *Ccl20-Luc* mice. [95] CCL20 (C–C motif chemokine ligand 20) is a CCL6 agonist that induces immune cell activation and chemotaxis. The purpose of the *Ccl20-Luc* mice was to unravel the organ-specific expression profile of *Ccl20* and monitor the immune activation when challenged with flagellin, a Toll-like receptor (TLR)^15^-5 agonist and CCL20 stimulant. The transgenesis required cloning of a *Ccl20* gene promoter within the firefly luciferase vector pGL-3 to form a *Ccl20-Luc* construct. Following that, the *Ccl20-Luc* vector was cloned with a downstream insert, internal ribosome entry site (IRES)-enhanced yellow fluorescent protein (EYFP),^16^ creating a reporter plasmid: *Ccl20-Luc-IRES-EYFP*. The reporter plasmid was introduced into the fertilized eggs of C57BL/6 J mice via microinjection. The TG mice were then injected intravenously/intraperitoneally (i.v/i.p) with the stimulant flagellin, followed by D-luciferin, to measure *in vivo* generated luminescence from different organs at multiple time points. The authors reported an insignificant expression of *Ccl20* in the blood, the duodenum, and the jejunum while bioluminescence was restricted to the upper abdomen in the challenged mice. The liver was found to generate the highest transcript of *Ccl20-*mRNA, which was equally reflected by bioluminescence.

The CXCL12 (C-X-C motif chemokine ligand 12) ligand binds to its receptor CXCR4, which then relays a stimulatory signal within the receptor-harboring cell for initiating chemotaxis or inducing inflammation. Sun et al. by generating CXCR4 knock-out mice [96] demonstrated the importance of CXCR4 receptor interaction with the cytokine CXCL12 with skin-resident NK T cells. In addition, they were able to demonstrate that deletion of this receptor would affect immune cell activation and the dynamics of chemotactic infiltration. The transgenics involved crossing CXCR4-floxed mice with ZBTB16-GFP/Cre reporter mice, generating CXCR4^fl/fl^-ZBTB16^cre/+^ progeny. ZBTB16, a positive selection marker of NK T cell precursor, marked skin-resident NK T cells by driving downstream GFP and Cre transgene. Furthermore, Cre expression would render skin-resident NK T cells devoid of CXCR4, thus preventing GFP emission. Next, skin-resident NK T cells from CXCR4^fl/fl^-ZBTB16^cre/+^ mice were adoptively transferred to CXCL12-DsRed recipient mice, which then were challenged to a skin inflammation inducer, DNFB (dinitrofluorobenzene). Intravital imaging revealed that the absence of CXCR4 led to reduced inflammation and minimal skin-resident NK T cell infiltration to the site and significantly lowered the production of IFN-γ in comparison to the mice administered with CXCR4 + GFP expressing SRN T cells. [96]

### Transgenic Mouse Models to Monitor Cytokines: Interferons

An interesting mouse model has been developed by Reynolds and colleagues: a novel bioluminescent IFN-γ reporting TG mice, designated as Gammaglow. [97] Gammaglow mice were tested for reporting IFN-γ in a long-term *in vivo* setting, where the mice were challenged to an infection or tumor or even during autoimmune reaction. At its core, the Gammaglow reporter construct consisted of a BAC clone vector where exons 1–4 of IFN-γ gene were replaced with a cassette composed of firefly luciferase followed by a GFP. The homology arm extending from exon 1 and exon 4 ensured correct recombination with the *Infg* gene. Lastly, the founder TG mouse was backcrossed several generations with C57BL/6 to derive a stable transgenic reporter expressing line. Primary immune response induced by immunization of the Gammaglow mouse revealed a periodic expression of IFN-γ. OprF (an antigen derived from *Pseudomonas aeruginosa*) in the presence of an adjuvant was injected at different doses in the footpads of the mice. The immunization triggered an adaptive T cell response by the generation of IFN-γ which was translated as a bioluminescent signal emission recorded over 11 days. At a priming dose of 25 µg, the highest average radiance was observed increasing from day 7 to day 9 and descending at day 11 post immunization. A similar trend of periodic signal emission was observed with a one-fifth (5 µg) priming dose of OprF. Lastly, the authors crossed Foxp3-DTR mouse model with Gammaglow mice to study autoimmune T cell induction in the absence of Foxp3 + Tregs. Following DT administration, the authors could then visualize a significant difference in bioluminescence emission peaking at day 6 in IFN-γ* Foxp3-DTR reporter mice when compared to non-DT administered mice.

### Transgenic Mouse Models to Monitor Cytokines: Interleukins

IL^17^ as interleukin, here IL-12, is composed of IL-12p35 and IL-12p40 subunits and is secreted post TLR stimulation from macrophages and dendritic cells (DCs). Binding of the agonist IL-12 to the IL-12 receptor complex (composed of IL-12R-β1 and IL12R-β2) induces T cell maturation and generation of IFN-γ and tumor necrosis factor-alpha (TNF-α) from T cells and NK cells. [98] Acknowledging the concomitant role of IL-12 in immune cell activation and response, a knock-in TG mouse, consisting of a bicistronic p40 gene expression through fluorescence, was developed by Reinhardt et al. [99] The transgenesis involved the insertion of a bicistronic cassette within exon 8 of murine 129/Sv *IL-12p40* genomic BAC clone. The bicistronic cassette consisted of a modified IRES element-linked upstream of EYFP gene followed by a bovine growth hormone polyadenylation signal and ending with loxP-floxed neomycin resistance segment. In situ p40 induction, post bacterial lipopolysaccharide (LPS)^18^ challenge, triggered EYFP expression reflecting p40 expression kinetics and localization in draining lymph nodes. Lastly, the authors were successful in demonstrating the migratory advantage of stimulated (CpG-oligodeoxynucleotides and OVA exposed) BMDCs (bone marrow-derived dendritic cells) expressing p40. In addition, the presence of p40 expressing DCs attracted a greater number of OVA-specific T cells secreting IL-12 in the draining lymph nodes in comparison to non-expressing control DCs.

An IL-1β-driven firefly luciferase-expressing TG mice [100] was developed by Li et al., which could non-invasively report IL-1β expression during inflammation. Although the bioluminescence emission was detectable, a non-specific signal and the background noise were the downside as noticed in that TG model. With the least interfering background noise and signal, Iwawaki et al. developed IL-1β-based dual-operating luciferase (IDOL) gene TG mice. [101] A newly generated IDOL TG mouse model proved advantageous due to its dual regulation during transcriptional and post-translational processing. When the authors intraperitoneally injected the combination of d-galactosamine/LPS to induce acute hepatitis or caerulein/LPS to induce pancreatitis, the ex vivo image analysis reflected signal emission from the expected organs, thus marking the TG model reliable to report tissue-specific inflammation in addition to systemic inflammation.

In the same year, Hayashi et al. generated *hIL-6*-BAC-*Luc* (WIM-6) TG mice. [102] Within the first exon of the IL-6 locus, firefly luciferase was introduced through homologous recombination. This gene cassette was then introduced into a plasmid expressing loxP-floxed neomycin resistance gene. Arabinose-induced FLP (flippase), exhibiting its recombinase activity from earlier BAC construct, would then remove the neomycin resistance cassette generating the finalized targeting construct. Inflammation-induced IL-6 would drive the luciferase, which, in the presence of luciferin, would emit bioluminescence. When the WIM-6 mice were challenged under the LPS infection model, IL-6-driven luciferase would emit bioluminescent signals, which in turn would reflect a tissue-distributed inflammatory situation. The authors could perform non-invasive, long-term visualization of inflammation in a mouse model of atopic dermatitis. To do so, AhR-CA TG mice that suffered from inflammatory skin lesions (dermatitis), starting from the weaning stage, were crossed with their own WIM-6 TG mice. The derived progeny exhibited IL-6-driven bioluminescence from week 4 onwards and the emission peaking around week 12 was evident with the spreading of dermatitis. Lastly, when dexamethasone (glucocorticoid receptor agonist) was dermally applied as an anti-inflammatory therapy, the authors observed a significant drop in IL-6 expression. Decreasing luminescence from week 8 to the lowest detected at week 11 reflected that drop, therefore exhibiting the applicability of *hIL6*-BAC-*Luc* WIM-6 TG mice not only to study IL-6 kinetics chronic inflammation but also for anti-inflammatory studies.

### Transgenic Mouse Models for Myeloid-Derived Cell Function: Monocyte/Macrophages

Within bone marrow hematopoiesis, common myeloid progenitor cells give rise to myeloblasts which later generate granulocytes which are neutrophils, basophils, and eosinophils produced via granulopoiesis, while monocytes give rise to macrophages and myeloid DCs through monocytopoiesis. Within these subtypes, the monocytic successors always intrigue researchers due to their unforeseen behavior in different tissue microenvironments. For monocytic macrophage’s recruitment and spatiotemporal distribution in mouse models for spinal cord injury (SCI),^19^ the groups of Mawhinney et al. and Fenrich et al. adapted and designed specific TG reporter mice. To start with, the TG mouse model used by Mawhinney and colleagues [103] helped them distinguish the origin of macrophages encircling SCI, i.e., if they originated from the blood or the central nervous system (CNS).^20^ The authors adapted *lys*-EGFP-*ki* TG mice developed by Faust et al. [104] The transgenics of *lys*-EGFP-*ki* involved the use of the *Lys* gene locus isolated from 129/Sv mouse genomic library. The novel targeting construct was sub-cloned within exon 1 of the lys gene such that the lys gene had an immediate EGFP which reported its expression. Based on lys-driven EGFP expression, Mawhinney and colleagues performed quantitative immunohistochemistry and analyzed flow cytometry data from the *lys*-EGFP-*ki* mice. The results gave a significant spatial and temporal distinction between hematogenous macrophages and microglia macrophages. Additionally, they were successful in demonstrating neutrophil dependence to hematogenous macrophages expressing EGFP for their subsistence around SCI. Although this mouse model has not yet been used for imaging, it is suited for intravital imaging and could provide a detailed insight *in vivo*. In the following year, Fenrich et al. [105] utilized an optical glass window mediated by two-photon microscopy for *in vivo* imaging. The imaging was performed to firstly observe myelomonocytic cell subset migration and interaction within the spinal cord lesion and secondly to investigate the role they perform during injury. To demonstrate this, the authors generated triple transgenic mice expressing Thy1-CFP//LysM-GFP//CD11c-EYFP which are capable of identifying multiple fluorescent cell populations. Three independent TG mouse models were used as a source to generate the desired TG mice. The first was from Feng et al. [106], Thy1 promoter-driven cyan fluorescent protein-23 mice that reported different neuron subsets. The second was from Faust et al. [104], LysM that drives GFP mice, which highlighted peripheral myelomonocytic cells. The last was adapted from Lindquist et al., in which CD11c-driven EYFP mice report microglial subset, which have a major focus on DCs. [107] Backcrossing these three TG mice with C57/B16 mice followed by crossbreeding was performed in order to derive the pups. These TG pups reported different cell populations based on their cell-specific promoter-driving distinct fluorescence. Through long-term optical imaging, the authors could distinctly visualize spatiotemporal distribution, morphology, and functioning of LysM + myelomonocytic cells (granulocytes, circulating macrophage precursors, and activated infiltrating macrophages) and CD11c + spinal cord-resident microglial subset, before and after SCI, within or around the lesion.

In order to track the monocytes and their differentiation into macrophages *in vivo* during inflammation, Iqbal et al. developed TG CD68-GFP reporter mice. [108] In these mice, human CD68 gene promoter drove EGFP expression, which was reported not only in the monocyte-derived macrophages within the blood, in the spleen, and in the bone marrow but also in the embryo-derived tissue-resident macrophages. The genetic modulation involved the sub-cloning of EGFP fragment downstream of human CD68 promoter. To highlight, from stage 8.5 onwards of the embryonic development, the authors were able to visualize monocytes/macrophages in the yolk sac around the periphery of the vasculature, sprouting blood vessels and embryo in the later stages. In terms of applicability, the authors found the monocytes from CD68-GFP reporter mice when adoptively transferred into recipient mice, with sterile inflammation, and the monocytes retained high GFP expression even after differentiation into macrophages. Lastly, to study monocyte trafficking and differentiation in chronic inflammation, intranasal bacille Calmette-Guérin (BCG) infection was conducted in the mice. Their results revealed that in immunofluorescence compared to that in the W.T mice, the infected mice had dense GFP + alveolar macrophages localizing at the site of infection. Additionally, localized macrophages were of M1 type (inflammatory and anti-microbial).

Subsequently, Evrard et al. [109] worked on *in vivo* visualization of the mobilizing bone marrow-derived monocyte using multiphoton intravital microscopy. The authors disapproved the *Cx3cr1*^*gfp/*+^ transgenic line as a favorable model for studying monocytes through intravital microscopy. They explained that *Cx3cr1*-driven GFP fluorescence from DCs would constantly interfere with that from the monocytes. This could potentially reduce the signal accuracy and reliability of *Cx3cr1*^*gfp/*+^ TG mice as a reporter of monocytes. Thus, to deplete GFP radiating DCs, the authors developed TG mice with *Flt3L*^*−/−*^ knock-out, while GFP is still retained within the monocytes. By doing so, the authors adapted two TG mouse models, one being *Flt3L*^*−/−*^ from Taconic Biosciences (Hudson, NY, USA) and *Cx3cr1*^*gfp*^ mice from The Jackson Laboratory (Bar Harbor, ME, USA). Crossbreeding these TG mice generated *Cx3cr1*^*gfp/*+^*Flt3L*^*−/−*^ reporter founders. [109] Intravital imaging was performed in the bone marrow of the skull by incorporating an optical window on the skull. Intravital imaging displayed a significant reduction in cells exhibiting DC morphology in comparison to the *Cx3cr1*^*gfp/*+^*Flt3L*^+*/*+^ control mice. Lastly, long-term monitoring of medullar and circulating Ly6C^hi^ monocyte population after bacterial LPS challenge proved that *Flt3L*^*−/−*^ did not interfere with Ly6C^hi^ monocyte emigration from B.M into the bloodstream.

Moving on from fluorescence to bioluminescence as a tool for *in vivo* reporting tumor-associated macrophages, He et al. generated LysM-LG TG mice [110] by crossing B6.129P2-Lyz2^tm1(cre)Ifo^/J mice (The Jackson Laboratory, ME, USA) with their own generated Cre-lox Luc reporter mice. In the former mice, Cre recombinase expression was driven by lysozyme M promoter while the latter had a dual reporter construct. To make the reporter construct, the gene cassette of the click beetle luciferase and *Heteractis magnifica* GFP was cloned downstream of loxP-floxed *Renilla luciferase* (rLuc), which had a stop codon after rLuc. The construct had a constitutively active hybrid chicken β-actin promoter and a CMV enhancer. The reporter construct was then microinjected in the oocyte of the recipient mice. Functionally, the derived LysM-LG TG pups from the crossing possessed CBLuc and GFP positive myeloid cells as the loxP-floxed RLuc segment was deleted through LysM-driven Cre. When LysM-LG mice were challenged with LPS, the authors reported increased click beetle luciferase signal, reflecting macrophage activation, which, later by flow cytometry, proved that macrophages were predominantly of M1 subtype, based on the markers expressed. When challenged i.p with murine ovarian cancer (ID8) and assessed longitudinally through bioluminescence imaging, the authors reported a similar increase in bioluminescence signal specifically at week 1 and week 12, reflecting the macrophage reporting efficiency over time.

### Transgenic Mouse Model for Monitoring Microglia

Microglial cells, also referred to as macrophages of the CNS, contribute to the reparations during neuronal damage and regulating neuronal homeostasis. [111] Furthermore, a study confirmed that they originate from primitive macrophages associated from yolk sac, which prevails in the CNS following adulthood. [112] In order to visualize microglia *in vivo*, Hirasawa et al. developed TG microglia Iba1-EGFP reporter mice. [113] *Iba1* was selected as a microglial-specific gene to be a fluorescent reporter using EGFP. The authors cloned the human *Iba1* gene as a promoter (with first two exons) from 129 Sv/J mouse cosmid library. To note, an IFN-γ-responsive element is present upstream of the second exon that contributes to Iba1-enhanced expression in the microglial cells. Furthermore, the reporter transgene was constructed by sub-cloning EGFP cDNA with an *Iba1* gene cassette into a novel backbone vector. When comparing the illuminated brain section of an adult TG mouse with that of non-TG mouse, the authors could visualize microglial cell-associated strong diffused fluorescence. Lastly, due to Iba1-EGFP reporter activity, the authors could monitor the spatiotemporal organization of microglial cells in developing mice embryos from day E10.5.

### Transgenic Mouse Models for Monitoring Dendritic Cell Function

Dendritic cells play an important role in initiating immune response by maturing (mDC) against foreign antigen/inflammatory mediators or maintaining tolerance against self-antigens by remaining as immature DCs (imDC). This phenomenon is achieved by processing and presenting antigens on their surface for other immune cells to recognize and respond to. [114, 115] To visualize these mDC and imDC deep in lymph nodes using two-photon fluorescence microscopy, Lindquist and colleagues developed CD11c-EYFP TG mice. ^(107)^ DC-specific CD11c promoter drove EYFP-Venus (adapted from Nagai et al.) [116] expression. The authors selected founders that exhibited the highest fluorescence tissue penetration (175–350 µm) and named them CD11c-EYFP^hi^ mice. 3D image reconstruction of inguinal lymph nodes using two-photon fluorescence microscopy revealed spatiotemporal kinetics of perifollicular and deeper CD11c-EYFP expressing DCs. In addition, their proximity to immune cells in subcapsular T-zone and T-B-interface compartment was also visualized. Lastly, the authors visualized mDC interaction with endogenous lymph node network. DCs were matured *in vitro* by exposing them to LPS, which then were injected intradermally into the flanks. Under periodic observation using intravital microscopy, the authors observed migration of LPS-matured CD11c-EYFP + DCs settling in the lymph nodes around B and T cell zones which at later time points (96 h) showed a decline in their numbers. From this observation, the authors conferred that mDCs are more motile than the steady-state DCs.

When CD11c-EYFP (B6.Cg-Tg (Itgax-EYFP) 1 Mnz/J) mice were administered with transcutaneous immunization, Rattanapak et al. could visualize in real-time the interaction between dermal DCs (DDCs) and immunogenic antigens using two-photon microscopy. [117] The authors favored the transcutaneous immunization because of the high numbers of antigen presenting DDCs and Langerhans cells (LCs) in the dermis and the microneedles could penetrate the stratum corneum so to deliver the immunogenic antigens intradermally. In the course of visualizing DC-immunogen interaction, it was revealed that the primary cells interacting with the immunogen were the DDCs displaying immediate mobility. In addition, the authors found an increase in their numbers over time in comparison to LCs. DDCs were distinctly recognizable from LCs due to their morphological differences and mobility characteristics. Importantly, under XY cross-sectional image analysis, the authors visualized DDCs extending into the epidermis for capturing and uptaking immunogens.

## Future Perspectives

As a common observation, generating a TG mouse involves detailed cloning strategies for assisting reporter gene cassettes to recombine within the host genome. This in turn, being challenging and time-consuming, does not ensure always the desired transgene expression and phenotype. Henceforth, moving towards optimizing pre-existing recombination methodology or testing enhanced mutants for site-specific knock-in insertions or adapting novel proteins involved in recombination events from a non-traditional organism to the bench would transform classical transgenics to become more efficient, replicable, and desirable. In addition, from the literature cited here, we observed that most of the TG mice developed for exploring immune cell function belonged to a particular subtype. For example, TG for visualizing B cell functionality was lacking and only a CD19-mCherry/luciferase transgenic is available to achieve B cell-restricted fluorescence/bioluminescence in living mice. Presumably, this might be due to difficulty generating a unique TG mouse that reports activated B cell functionality or localization. Lastly, combining different reporter genes within TG targeting gene would render it to be visualized using multi-modality reporters. A notable example would be the use of optoacoustic gene combined with near-infrared-emitting luciferase for reporting immune cell functionality within deep tissue. We expect that future transgenic mouse models for monitoring gene expression dynamics in immune cells would make use of different imaging modalities so that sensitivity and versatility of the models would be increased.

## Footnotes

1 (TG) transgenic.

2 (NK) natural killer.

3 CRISPR-Cas (clustered regularly interspaced short palindromic repeats-cellular apoptosis susceptibility).

4 (Cre) cyclization recombinase.

5 (IVM) intravital microscopy.

6 (EGFP) enhanced green fluorescent protein.

7 (DsRed) *Discosoma* sp. red fluorescent protein.

8 (nTreg) naïve regulatory T-cells.

9 (Teff) effector T-cells.

10 (BAC) bacterial artificial chromosome.

11 (CBG99) click beetle green luciferase.

12 (NFAT) nuclear factor of activated T cells.

13 (CBR) click beetle red luciferase.

14 (INF) interferon.

15 (TLR) Toll-like receptor.

16 (EYFP) enhanced yellow fluorescent protein.

17 (IL) interleukin.

18 (LPS) lipopolysaccharides.

19 (SCI) spinal cord injury.

20 (CNS) central nervous system.
