# Correction: Monitoring Immune Cell Function Through Optical Imaging: a Review Highlighting Transgenic Mouse Models

**DOI:** 10.1007/s11307-024-01930-0

**Published:** 2024-07-12

**Authors:** Chintan Chawda, Roisin McMorrow, Natasa Gaspar, Giorgia Zambito, Laura Mezzanotte

**Affiliations:** 1 Department of Radiology and Nuclear Medicine, Erasmus MC, Rotterdam, The Netherlands; 2 Department of Molecular Genetics, Erasmus MC, Rotterdam, The Netherlands; 3 Percuros B.V, Leiden, The Netherlands

**Correction: Molecular Imaging and Biology (2021) 24:250-263**

10.1007/s11307-021-01662-5

The original online version of this article was revised to add the following funding information:

This work was supported by the European Commission under the European Marie Skłodowska-Curie Innovative Training Network (ITN) scheme (Grant Agreement number: 813834 and Grant Agreement number: 861190).

The original article has been corrected.

